# A match-day analysis of the movement profiles of substitutes from a professional soccer club before and after pitch-entry

**DOI:** 10.1371/journal.pone.0211563

**Published:** 2019-01-31

**Authors:** Samuel P. Hills, Steve Barrett, Richard G. Feltbower, Martin J. Barwood, Jon N. Radcliffe, Carlton B. Cooke, Liam P. Kilduff, Christian J. Cook, Mark Russell

**Affiliations:** 1 School of Social and Health Sciences, Leeds Trinity University, Leeds, United Kingdom; 2 Sport Medicine and Science Department, Hull City Tigers FC, Kingston Upon Hull, United Kingdom; 3 Department of Clinical & Population Sciences, School of Medicine, University of Leeds, Leeds, United Kingdom; 4 Applied Sports Technology, Exercise Medicine Research Centre (A-STEM), Swansea University, Swansea, United Kingdom; 5 Welsh Institute of Performance Science, College of Engineering, Swansea University, Swansea, United Kingdom; 6 UC Research Institute for Sport & Exercise, University of Canberra, Canberra, Australia; Nottingham Trent University, UNITED KINGDOM

## Abstract

Whilst the movement demands of players completing a whole soccer match have been well-documented, comparable information relating to substitutes is sparse. Therefore, this study profiled the match-day physical activities performed by soccer substitutes, focusing separately on the pre and post pitch-entry periods. Seventeen English Championship soccer players were monitored using 10 Hz Micromechanical Electrical Systems (MEMS) devices during 13 matches in which they participated as substitutes (35 observations). Twenty physical variables were examined and data were organised by bouts of warm-up activity (pre pitch-entry), and five min epochs of match-play (post pitch-entry). Linear mixed modelling assessed the influence of time (i.e., ‘bout’ and ‘epoch’), playing position, and match scoreline. Substitutes performed 3±1 rewarm-up bouts∙player^-1^∙match^-1^. Compared to the initial warm-up, each rewarm-up was shorter (-19.7 to -22.9 min) and elicited less distance (-606 to -741 m), whilst relative total distances were higher (+26 to +69 m∙min^-1^). Relative total (+13.4 m∙min^-1^) and high-speed (+0.4 m∙min^-1^) distances covered during rewarm-ups increased (p <0.001) with proximity to pitch-entry. Players covered more (+3.2 m; p = 0.047) high-speed distance per rewarm-up when the assessed team was losing compared with when winning at the time of pitch-entry. For 10 out of 20 variables measured after pitch-entry, values reduced from 0–5 min thereafter, and substitutes covered greater (p ˂0.05) total (+67 to +93 m) and high-speed (+14 to +33 m) distances during the first five min of match-play versus all subsequent epochs. Midfielders covered more distance (+41 m) per five min epoch than both attackers (p ˂0.001) and defenders (p = 0.016). Acknowledging the limitations of a solely movement data approach and the potential influence of other match-specific factors, such findings provide novel insights into the match-day demands faced by substitute soccer players. Future research opportunities exist to better understand the match-day practices of this population.

## Introduction

Soccer match-day squads include a number of substitutes, with whom managers may replace members of the starting team during a match [1]. English Football (soccer) League rules currently permit up to three substitutions from a maximum of six nominated players [2]. Substitutes are typically introduced to offset the effects of fatigue, change tactics, or replace injured/underperforming players [3]; although other motivations may exist (e.g., allowing playing time for youth players or those returning from injury: [4]). Whilst situational variables (i.e., league-type, match scoreline) may also influence substitute timing, substitutions typically occur after at least 45 min of match-play [3, 5], with midfielders being the most common replacement [5].

Soccer-specific exercise compromises indices of physical and technical performance throughout 90 min [6–8], responses which appear exacerbated during matches requiring extra-time [9–11]. Notably, for players starting a match, high-speed running (HSR; typically defined as moving at a speed ˃5.5 m∙s^-1^), a commonly-used indicator of physical performance in soccer; and one that may discriminate between playing levels [6], reduces progressively during match-play [6, 12]. As strategic substitutions often represent a means by which coaches/managers seek to attenuate fatigue-induced deteriorations in physical performance [3, 4], the efficacy of this strategy remains to be confirmed. Indeed, although other motivations may underpin the decision to make a replacement (e.g., technical/tactical considerations), it has been proposed that for a substitution to be deemed effective from a work-rate perspective, substitutes entering the field of play need to achieve or surpass the running speeds of players being replaced and/or remaining on the pitch [3].

English Premier League substitutes have demonstrated a trend towards increasing total distance (TD) and HSR over successive five min periods following introduction [3], thus possibly suggesting either conservative self-pacing strategies or questioning the efficacy of pre pitch-entry preparations. However, as the match epochs used for analysis were determined relative to kick-off (e.g., data from a player introduced at 57 min would not register until the next five min epoch; 60–65 min), such responses could have been influenced by the potential omission of the moments immediately following players’ introduction. Conversely, substitutes entering the pitch at half-time or during the second-half appear able to exceed the relative (m∙min^-1^) TD and HSR performed by players who started a match (i.e., players remaining on the pitch or those being replaced: [3, 6, 13]). Whilst substitutes may perform more HSR than during the equivalent second-half period when the same players complete a full-match, they appear unable to exceed the HSR performed during the first-half of matches that they start [3, 13, 14], despite it being assumed that substitutes enter the pitch in a state free from substantially accumulated acute fatigue. Acknowledging the likely influence of match-specific contextual variables (e.g., scoreline, opposition quality, potential differences in playing formation etc.) on the movement profiles observed, such observations may call into question whether the pre pitch-entry strategies employed by soccer substitutes facilitate optimal performance following match introduction, especially given players’ and coaches’ desire for substitutes to make an immediate and sustained impact on the match [4].

Because substitutes typically face lengthy delays (often ≥75 min) between cessation of the initial warm-up (i.e., preparatory activities performed prior to kick-off) and their entry onto the pitch [3, 5], punctuated by only brief bouts of rewarm-up activity [4], their actions during this period are of particular interest if preparedness for match-introduction is to be optimised. However, despite the direct relevance for subsequent match performance, we are unaware of any study that has profiled the specific preparatory activities undertaken by soccer substitutes. Therefore, the dual objectives of this research were to investigate both substitutes’ pre pitch-entry activities, and their physical performance responses following introduction into the match.

## Materials and methods

### Experimental approach

Following specific project approval from the School of Social and Health Sciences sub-committee of the Leeds Trinity University ethics board (SSHS-2017-077), professional male players (n = 17; age: 25±8 years; stature: 1.80±0.09 m; body mass: 85.2±8.6 kg) from an English Championship soccer club (representing the second tier of professional soccer in the United Kingdom) were monitored throughout 13 home league matches in which they participated as substitutes during the latter half of the 2017/18 competitive season. Data represents those players who were introduced at half-time or during the second-half of a match (i.e., not from unused substitutes, or enforced injury replacements made during the first-half) and included three defenders, seven forwards and seven midfielders, who each undertook four football-specific and gym-based training sessions in addition to one-to-two 90 min matches per week. Given the observational nature of the study, no attempt was made to influence players’ responses, and activity monitoring was routinely required as part of their employment. Written informed consent was achieved and a total of 35 performance observations (16, 14, and 5 observations from midfielders, attackers, and defenders, respectively; 2±2 matches∙player^-1^; range: 1–6 matches∙player^-1^) were included.

### Activity monitoring

Players’ movements were captured by 10 Hz Micromechanical Electrical Systems (MEMS; S5, Optimeye; Catapult Innovations, Melbourne, Australia) units worn between the scapulae in a specifically designed vest. A combination of Global Positioning Systems and accelerometer-derived variables were profiled. MEMS sampling at 10 Hz have demonstrated acceptable reliability (coefficient of variation; CV% = 2.0–5.3%) for measuring instantaneous velocity [15], and the specific units used demonstrated small-to-moderate typical error of the estimate (1.87–1.95%) versus a radar gun when assessing sprinting speed [16]. Similarly, no significant differences between criterion values and MEMS-derived measures of TD were observed during a team sport-specific circuit [17], whilst very large or near-perfect correlations (r = 0.89–0.91) were reported for peak speed [17]. Notably, at all speeds examined (1–8 m∙s^-1^), CV% less than or similar to the smallest worthwhile change in performance (0.2 multiplied by the between-participant standard deviation [18]) have been observed for constant velocity, acceleration, and deceleration during straight line running [15]. The accelerometers within the devices have also demonstrated good intra (CV% = 0.9–1.1%) and inter-unit (CV% = 1.0–1.1) reliability in both laboratory and field test environments [19]. Players wore the same units in each match and were filmed (50 Hz; GX1; JVC; Yokohama; Japan) prior to pitch-entry.

In accordance with manufacturer’s guidelines, the MEMS units were activated outdoors and ~30 min prior to the initial warm-up, whilst raw data were exported post-match (Sprint 5.1.7, Catapult Innovations, Melbourne, Australia). Table 1 defines the MEMS-derived variables profiled. Data were organised on an individual player basis, and were classified into periods according to each bout of warm-up activity performed (pre pitch-entry) and into five min epochs from the moment a player entered the pitch (post pitch-entry). For each substitution, contextual information relating to match scoreline, playing position, and the timing of introduction was also recorded.

**Table 1:** Operational definition for Micromechanical Electrical Systems (MEMS)-derived outcome variables.

Measurement	Variable	Definition
**Distance covered**	Total (m)	Total amount of distance covered by any means
	Relative total (m∙min^-1^)	Total amount of distance covered per min
	Low-speed running (m)	Distance covered at a speed of ≤4 m∙s^-1^
	Relative low-speed running (m∙min^-1^)	Distance covered per min at a speed of ≤4 m∙s^-1^
	Moderate-speed running (m)	Distance covered at a speed of ˃4 to ≤5.5 m∙s^-1^
	Relative moderate-speed running (m∙min^-1^)	Distance covered per min at a speed of ˃4 to ≤5.5 m∙s^-1^
	High-speed running (m)	Distance covered at a speed of ˃5.5 to ≤7 m∙s^-1^
	Relative high-speed running (m∙min^-1^)	Distance covered per min at a speed of ˃5.5 to ≤7 m∙s^-1^
	Sprinting (m)	Distance covered at a speed of ˃7 m∙s^-1^
	Relative sprinting (m∙min^-1^)	Distance covered per min at a speed ˃7 m∙s^-1^
**Player Load**	Absolute (AU)	Quantification of external workload: Square root of the summed rates of change in instantaneous velocity in each of the three (forwards, sideways, upwards) vectors, divided by a scaling factor of 100
	Relative (AU∙min^-1^)	Player load accumulated over X number of min, divided by X number of min
**Acceleration/deceleration count**	High-intensity accelerations (#)	Count of the number of accelerations >3 m∙s^−2^ for a period of ≥0.4 s
	High-speed decelerations (#)	Count of the number of decelerations <−3 m∙s^−2^ for a period of ≥0.4 s
	Moderate-speed accelerations (#)	Count of the number of accelerations ˃2 to ≤3 m∙s^−2^ for a period of ≥0.4 s
	Moderate-speed decelerations (#)	Count of the number of decelerations ˂−2 to ≥−3 m∙s^−2^ for a period of ≥0.4 s
**Acceleration/deceleration distance**	High-speed acceleration (m)	Distance covered whilst accelerating at >3 m∙s^−2^
	High-speed deceleration (m)	Distance covered whilst decelerating at <−3 m∙s^−2^
	Moderate-speed acceleration (m)	Distance covered whilst accelerating at ˃2 to ≤3 m∙s^−2^
	Moderate-speed deceleration (m)	Distance covered whilst decelerating at <−2 to ≥−3 m∙s^−2^
**Time**	Duration (min)	Length of time for any given period

AU: Arbitrary units,

^#^: Count,

MEMS: Micromechanical Electrical Systems.

### Statistical analyses

To account for the interdependence of data arising through repeated observations across multiple matches, linear mixed modelling was conducted to differentiate outcome variables as a function of time. ‘Match’ and ‘player’ were entered as random effects, whilst playing position and match scoreline at the time of introduction were specified as fixed categorical variables. Time (i.e., ‘epoch’ or ‘bout’) was modelled first as a continuous, and then categorical (fixed) variable to allow comparisons with a baseline reference, for which the first time-period (i.e. initial warm-up or 0–5 min for pre and post pitch-entry data, respectively) was used. For the fixed effect of position, midfielders were used as baseline, whilst for the scoreline variable, the team being ahead in a match was specified as the reference category. For each outcome measure, a variance components model with no predictors was established before sequentially allowing intercepts and then slopes to vary. A combination of random slopes and intercepts were employed based upon Bayesian information criterion assessments of model fit. For ‘count’ data, responses were transformed to the incidence rate ratio (IRR) scale and analysed via mixed-effects Poisson regression. Analyses were conducted using StataCorp; 2017, Stata Statistical Software Release 15, College Station, TX: StataCorp LLC. Data below are presented as mean±standard deviation (SD), whilst magnitude of change is demonstrated by effect estimates (or IRR for ‘count’ variables), with associated 95% confidence intervals.

## Results

All three replacements were utilised in 12 out of 13 matches and the mean timing of the first, second and third substitutions were 59±9, 71±10, and 77±10 min, respectively. Video footage indicted that substitutes’ initial (pre-match) warm-ups were conducted separately from the starting players and began with dynamic stretching (~10 min) followed by possession games (~10 min) and passing sequences (~6 min) before returning to the changing rooms ~15 min before kick-off. The initial warm-up remained consistent across all matches profiled. Following kick-off, substitutes mostly remained seated; occasionally standing to perform rewarm-up activity (3±1 rewarm-up bouts∙player∙match^-1^). The team won four, drew three, and lost six of the 13 matches, scoring and conceding a total of 16 and 13 goals, respectively. In 13 of the 35 substitutions observed, a player entered the pitch when the team was leading (in terms of match scoreline) in the match. In a further 13 instances a substitution was made when the team was behind, whilst the remaining nine substitutes were introduced when the scores were level. The mean scoreline was 1±1 goal scored and 1±1 goal conceded at the time of pitch-entry for each of the first, second, and third substitutions, respectively. There were nine occasions when the team goal differential (i.e., goals scored minus goals conceded) improved during the time between a substitution being made and the end of the match. The goal differential became less favourable following seven of the substitutions, and 19 instances were observed in which the difference between the two teams was the same after 90 min when compared with the time of pitch-entry.

Tables 2 and 3 detail the pre pitch-entry activities performed. Each rewarm-up was shorter than the initial warm-up, and TD, Player load (PL) low-speed running distances (LSR) and the number of moderate-speed accelerations (#M_ACC_) were also lower for rewarm-ups. However, relative TD, PL, and LSR during each rewarm-up exceeded initial warm-up values. Relative HSR during the first (p = 0.345) and half-time (p = 0.194) rewarm-up bouts was similar to the initial warm-up, but relative HSR was significantly higher for all second-half rewarm-ups compared with the initial warm-up. Effect estimates indicated significant decreases in duration, absolute TD, absolute LSR, and absolute PL for each successive bout of pre pitch-entry activity performed. However, increases were observed for absolute and relative moderate-speed running distance (MSR) and HSR, relative TD, relative LSR, and relative PL, as well as distance covered whilst decelerating at high (H_DEC_dist), and moderate (M_DEC_dist) speeds. In addition, increases in the number of #H_ACC_, #M_ACC_, #H_DEC_, and #M_DEC_ were observed as proximity to pitch-entry neared. Scoreline at the time of pitch-entry influenced the amount HSR performed during rewarm-up activity, with players covering an additional 3.17 m of HSR per rewarm-up bout (p = 0.047, CI: +0.04 to +6.31 m) when the team was losing at the time of introduction, when compared with when the team was ahead. Scoreline did not influence any other variable prior to pitch-entry.

**Table 2:** Descriptive statistics for physical performance variables for substitutes prior to pitch-entry.

Variable		Initial warm-up (*n = 35*)	RWU1 (*n = 34 first-half*, *n = 1 second-half*)	Half-time warm-up (*n = 27*)	RWU2 (*n = 6 first-half*, *n = 22 second-half*)	RWU3 (*n = 1 first-half*, *n = 7 second-half*)	RWU4 (*n = 2 second-half*,)
**Duration (min)**		26.25 ± 2.43	6.51 ± 2.39^b^	5.51 ± 2.31^b^	5.96 ± 3.74^b^	3.14 ± 1.68^b^	3.23 ± 0.39^b^
**TD**	Absolute (m)	992 ± 218	386 ± 143^b^	423 ± 170^b^	428 ± 286^b^	229 ± 93^b^	321 ± 44^b^
	Relative (m∙min^-1^)	37.9 ± 7.8	64.3 ± 23.5^b^	83.0 ± 30.3^b^	80.2 ± 28.9^b^	89.3 ± 40.2^b^	99.5 ± 1.6^b^
**LSR**	Absolute (m)	963 ± 210	369 ± 131^b^	394 ± 159^b^	378 ± 259^b^	198 ± 100^b^	280 ± 45^b^
	Relative (m∙min^-1^)	36.8 ± 7.5	61.1 ± 19.8^b^	76.1 ± 22.9^b^	70.7 ± 25.6^b^	72.3 ± 28.1^b^	86.5 ± 3.4^b^
**MSR**	Absolute (m)	15 ± 31	15 ± 22	18 ± 28	42 ± 39^b^	27 ± 26	37 ± 6
	Relative (m∙min^-1^)	0.6 ± 1.2	2.9 ± 5.5	4.5 ± 8.7^a^	8.6 ± 9.3^b^	14.7 ± 18.0^b^	11.3 ± 0.6^b^
**HSR**	Absolute (m)	1 ± 4	2 ± 6	3 ± 6	6 ± 10^a^	3 ± 5	5 ± 7
	Relative (m∙min^-1^)	0.0 ± 0.1	0.3 ± 1.0	0.5 ± 1.3	0.8 ± 1.2^a^	1.9 ± 3.9^b^	1.7 ± 2.4^a^
**SPR**	Absolute (m)	0 ± 0	0 ± 0	0 ± 0	0 ± 0	0 ± 0	0 ± 0
	Relative (m∙min^-1^)	0.0 ± 0.0	0.0 ± 0.0	0.0 ± 0.0	0.0 ± 0.0	0.0 ± 0.0	0.0 ± 0.0
**PL**	Absolute (AU)	127.64 ± 24.10	38.54 ± 12.56^b^	40.19 ± 19.29^b^	42.50 ± 27.31^b^	20.54 ± 9.26^b^	30.27 ± 1.87^b^
	Relative (AU∙min^-1^)	4.88 ± 0.90	6.58 ± 2.79 ^b^	7.54 ± 2.05^b^	7.90 ± 2.77^b^	7.82 ± 3.72^b^	9.42 ± 0.55^b^
**_ACC_dist**	High (m)	2 ± 2	1 ± 1^a^	2 ± 3	1 ± 4	1 ± 1	1 ± 0
	Moderate (m)	7 ± 4	3 ± 3^b^	5 ± 4	6 ± 7	2 ± 1	7 ± 1
**_DEC_dist**	High (m)	0 ± 0	0 ± 1	0 ± 1	1 ± 1^b^	0 ± 0	0 ± 0
	Moderate (m)	1 ± 2	1 ± 2	2 ± 2	4 ± 4^b^	1 ± 1	2 ± 1
**#_ACC_**	High (#)	2 ± 2	1 ± 1^b^	2 ± 2	1 ± 2^b^	0 ± 1^a^	0 ± 0
	Moderate (#)	11 ± 6	3 ± 2^b^	6 ± 4^b^	4 ± 4^b^	1 ± 1^b^	3 ± 1^b^
**#_DEC_**	High (#)	0 ± 1	0 ± 1	1 ± 1	1 ± 1^b^	0 ± 1	1 ± 1
	Moderate (#)	3 ± 2	1 ± 2^a^	2 ± 2	3 ± 3	1 ± 1^a^	4 ± 2

_ACC_dist: Acceleration distance, AU: Arbitrary units, _DEC_dist: Deceleration distance, HSR: High-speed running, LSR: Low-speed running, MSR: Moderate-speed running, PL: Player Load, RWU: Rewarm-up, SPR: Sprinting, TD: Total Distance, #_ACC_: Number of accelerations, #_DEC_: Number of decelerations,

^a^ different from initial warm-up at p≤0.05 level,

^b^ different from initial warm-up at p≤0.001 level.

**Table 3:** Magnitude of change in physical performance variables for substitutes prior to pitch-entry.

Variable		Initial warm-up (*n = 35*)	RWU1 (*n = 34 first-half*, *n = 1 second-half*)	Half-time warm-up (*n = 27*)	RWU2 (*n = 6 first-half*, *n = 22 second-half*)	RWU3 (*n = 1 first-half*, *n = 7 second-half*)	RWU4 (*n = 2 second-half*,)	Time effects
**Duration**	(min)	REF	-19.74 (-20.92 to -18.55)^b^	-20.79 (-22.06 to -19.51)^b^	-20.30 (-21.56 to -19.04)^b^	-22.88 (-24.86 to -20.90)^b^	-22.86 (-26.56 to -19.15)^b^	-5.30 (-6.12 to -4.47)**
**TD**	Absolute (m)	REF	-606.46 (-692.96 to -519.95)^b^	-572.23 (-665.70 to -478.76)^b^	-565.32 (-657.26 to -473.38)^b^	-740.62 (-884.96 to -596.28)^b^	-642.28 (-914.96 to -369.60)^b^	-152.12 (-186.51 to -117.73)**
	Relative (m∙min^-1^)	REF	26.44 (15.94 to 36.94)^b^	46.30 (34.97 to 57.63)^b^	42.69 (31.53 to 53.84)^b^	52.41 (34.95 to 69.88)^b^	68.88 (35.95 to 101.80)^b^	13.43 (10.31 to 6.55)**
**LSR**	Absolute (m)	REF	-594.51 (-674.55 to -514.48)^b^	-575.86 (-662.39 to -489.34)^b^	-586.62 (-671.70 to -501.53)^b^	-745.66 (-879.37 to -611.96)^b^	-669.70 (-922.48 to -416.92)^b^	-157.85 (-190.36 to -125.39)**
	Relative (m∙min^-1^)	REF	24.33 (15.92 to 32.73)^b^	40.03 (30.75 to 49.32)^b^	33.86 (24.30 to 43.42)^b^	32.71 (16.79 to 48.63)^b^	55.87 (25.26 to 86.49)^b^	10.18 (7.34 to 13.02)**
**MSR**	Absolute (m)	REF	0.17 (-12.05 to 12.39)	5.69 (-7.53 to 18.92)	27.92 (14.91 to 40.92)^b^	16.89 (-3.61 to 37.40)	31.94 (-6.81 to 70.69)	7.59 (4.10 to 11.09)**
	Relative (m∙min^-1^)	REF	2.36 (-0.57 to 5.29)	4.27 (0.87 to 7.66)^a^	8.02 (4.28 to 11.75)^b^	16.12 (9.79 to 22.45)^b^	20.48 (8.77 to 32.18)^b^	3.04 (1.93 to 4.14)**
**HSR**	Absolute (m)	REF	0.83 (-1.71 to 3.37)	1.67 (-1.40 to 4.74)	4.62 (1.06 to 8.18)^a^	5.76 (-0.18 to 11.71)	9.10 (-1.45 to 19.66)	1.44 (0.40 to 2.49)*
	Relative (m∙min^-1^)	REF	0.24 (-0.26 to 0.74)	0.39 (-0.20 to 0.98)	0.78 (0.12 to 1.44)^a^	2.38 (1.26 to 3.49)^b^	3.00 (0.96 to 5.03)^a^	0.35 (0.16 to 0.54)**
**SPR**	Absolute (m)	REF	n/a	n/a	n/a	n/a	n/a	n/a
	Relative (m∙min^-1^)	REF	n/a	n/a	n/a	n/a	n/a	n/a
**PL**	Absolute (AU)	REF	-89.10 (-97.81 to -80.39)^b^	-87.09 (-96.5 to -77.67)^b^	-84.89 (-94.14 to -75.63)^b^	-103.72 (-118.25 to -89.8)^b^	-94.20 (-121.66 to -66.73)^b^	-22.26 (-26.53 to -17.99)**
	Relative (AU∙min^-1^)	REF	1.70 (0.74 to 2.66)^b^	2.89 (1.85 to 3.92)^b^	3.08 (2.06 to 4.10)^b^	3.23 (1.62 to 4.83)^b^	5.42 (2.39 to 8.44)^b^	0.95 (0.67 to 1.22)**
**_ACC_dist**	High (m)	REF	-1.49 (-2.50 to -0.47)^a^	0.13 (-1.08 to 1.34)	-0.70 (-2.06 to 0.67)	-0.33 (-2.63 to 1.97)	-0.21 (-4.38 to 3.97)	n/a
	Moderate (m)	REF	-4.46 (-6.13 to -2.79)^b^	-1.78 (-3.83 to 0.27)	-1.16 (-3.57 to 1.26)	-2.65 (-6.65 to 1.35)	1.82 (-5.21 to 8.84)	n/a
**_DEC_dist**	High (m)	REF	0.17 (-0.18 to 0.52)	0.25 (-0.16 to 0.65)	0.85 (0.41 to 1.30)^b^	0.50 (-0.26 to 1.25)	0.39 (-1.01 to 1.78)	0.21 (0.08 to 0.35)*
	Moderate (m)	REF	0.03 (-0.96 to 1.02)	0.50 (-0.66 to 1.65)	2.23 (0.95 to 3.52)^b^	0.15 (-2.02 to 2.33)	0.87 (-3.12 to 4.86)	0.51 (0.12 to 0.91)*
**#_ACC_**	High (IRR)	REF	0.32 (0.20 to 0.50)^b^	0.73 (0.50 to 1.06)	0.32 (0.19 to 0.52)^b^	0.18 (0.06 to 0.58)^a^	n/a	1.61 (1.54 to 1.69)**
	Moderate (IRR)	REF	0.24 (0.19 to 0.31)^b^	0.53 (0.43 to 0.65)^b^	0.41 (0.33 to 0.52)^b^	0.15 (0.08 to 0.26)^b^	0.21 (0.09 to 0.53)^b^	1.50 (1.42 to 1.58)**
**#_DEC_**	High (IRR)	REF	1.1 (0.47 to 2.59)	1.91 (0.85 to 4.32)	3.46 (1.69 to 7.16)^b^	1.23 (0.34 to 4.51)	1.92 (0.24 to 15.30)	2.30 (2.15 to 2.47)**
	Moderate (IRR)	REF	0.58 (0.41 to 0.82)^a^	0.86 (0.62 to 1.20)	1.09 (0.80 to 1.48)	0.27 (0.12 to 0.62)^a^	1.53 (0.69 to 3.41)	1.69 (1.62 to 1.77**

_ACC_dist:: Acceleration distance, AU: Arbitrary units, _DEC_dist: Deceleration distance, HSR: High-speed running, LSR: Low-speed running, MSR: Moderate-speed running, PL: Player Load, REF: Reference category for comparison, RWU: Rewarm-up, SPR: Sprinting, TD: Total Distance, #_ACC_: Number of accelerations, #_DEC_: Number of decelerations,

^a^ different from initial warm-up at p≤0.05 level when ‘bout’ modelled as categorical,

^b^ different from initial warm-up at p≤0.001 level when ‘bout’ modelled as categorical,

*: Significant effect at p≤0.05,

**: Significant effect at p≤0.001.

Data are reported as effect estimates (95% CI), except for #_ACC_ and #_DEC_ which are incidence risk ratios (IRR).

Tables 4 and 5 present activity profile data following substitutes’ introduction into a match. Effect estimates indicated significant declines in absolute and relative values for TD, MSR, HSR, and PL, as well as decreases in H_ACC_dist and M_ACC_dist from 0–5 min onwards as a function of time. Notably, 38.6% reductions in relative HSR (-3.94, CI: -5.77 to -2.11 m∙min^-1^), and 12.2% declines in both relative TD (-14.58, CI: -20.70 to -8.46 m∙min^-1^) and relative PL (-1.50, CI: -2.16 to -0.85 AU∙min^-1^) were observed from 0–5 min to 5–10 min. Moreover, 31.1% (-6.52, CI: -9.41 to -3.63 m∙min^-1^) and 20.0% (-3.22, CI: -5.03 to -1.41 m) decrements occurred for relative MSR and M_ACC_dist, respectively.

**Table 4:** Descriptive statistics for physical performance variables for substitutes from timing of pitch-entry to the end of match-play.

Variable		0–5 min (*n = 33*)	5–10 min (*n = 32*)	10–15 min (*n = 30*)	15–20 min (*n = 26*)	20–25 min (*n = 19*)	25–30 min (*n = 11*)	30–35 min (*n = 7*)	35–40 min (*n = 4*)
**TD**	Absolute (m)	599 ± 75	527 ± 66^b^	527 ± 81^b^	531 ± 59^b^	527 ± 60^b^	508 ± 72^b^	507 ± 110^b^	521 ± 56^a^
	Relative (m∙min^-1^)	120.0 ± 14.8	105.3 ± 13.3^b^	105.6 ± 16.5^b^	106.0 ± 11.5^b^	105.2 ± 11.8^b^	101.7 ± 14.5^b^	101.4 ± 22.1^b^	104.2 ± 11.2^a^
**LSR**	Absolute (m)	438 ± 55	414 ± 49	414 ± 66	413 ± 7	402 ± 48 ^a^	405 ± 51	425 ± 80	431 ± 58
	Relative (m∙min^-1^)	87.6 ± 11.0	82.9 ± 9.8	82.8 ± 13.2	82.5 ± 9.3	80.5 ± 9.5 ^a^	81.1 ± 10.3	84.9 ± 16.0	86.1 ± 11.5
**MSR**	Absolute (m)	105 ± 34	72 ± 27^b^	78 ± 38^b^	78 ± 29^b^	84 ± 36^a^	68 ± 33^b^	58 ± 28^b^	72 ± 29^a^
	Relative (m∙min^-1^)	20.9 ± 6.8	14.3 ± 5.4^b^	15.5 ± 7.5^b^	15.7 ± 5.8^b^	16.8 ± 7.1^a^	13.6 ± 6.6^b^	11.5 ± 5.5^b^	14.3 ± 5.8^a^
**HSR**	Absolute (m)	51 ± 29	31 ± 22^b^	28 ± 19^b^	30 ± 20^b^	36 ± 22^a^	24 ± 18^b^	20 ± 19^b^	18 ± 14^b^
	Relative (m∙min^-1^)	10.1 ± 5.9	6.2 ± 4.5^b^	5.7 ± 3.9^b^	6.1 ± 4.0^b^	7.1 ± 4.4^a^	4.9 ± 3.6^b^	3.9 ± 3.7^b^	3.7 ± 2.8^b^
**SPR**	Absolute (m)	6 ± 10	10 ± 15	7 ± 11	10 ± 12	5 ± 10	11 ± 14	5 ± 9	1 ± 2
	Relative (m∙min^-1^)	1.3 ± 1.9	2.1 ± 3.1	1.4 ± 2.1	2.0 ± 2.5	1.1 ± 2.0	2.2 ± 2.8	1.1 ± 1.8	0.2 ± 0.3
**PL**	Absolute (AU)	61.21 ± 8.43	53.94 ± 6.80^b^	52.78 ± 9.65^b^	53.04 ± 8.17^b^	52.90 ± 7.07^b^	49.67 ± 6.28^b^	46.31 ± 10.35^b^	45.76 ± 10.38 ^b^
	Relative (AU∙min^-1^)	12.25 ± 1.67	10.77 ± 1.39^b^	10.59 ± 1.94^b^	10.59 ± 1.61^b^	10.55 ± 1.41^b^	9.93 ± 1.25^b^	9.26 ± 2.07^b^	9.15 ± 2.08^b^
**_ACC_dist**	High (m)	8 ± 3	6 ± 3^a^	5 ± 3^b^	5 ± 3^b^	7 ± 4	6 ± 2	3 ± 3^b^	6 ± 4
	Moderate (m)	16 ± 5	13 ± 5^b^	13 ± 4^b^	13 ± 4^b^	14 ± 5^a^	13 ± 4^a^	10 ± 2^b^	11 ± 2^a^
**_DEC_dist**	High (m)	5 ± 2	4 ± 3 ^a^	3 ± 1 ^a^	4 ± 3	5 ± 3	4 ± 1^a^	3 ± 2^a^	3 ± 3 ^a^
	Moderate (m)	10 ± 4	8 ± 5	8 ± 3 ^a^	8 ± 3	8 ± 4	8 ± 3	7 ± 4	7 ± 4
**#_ACC_**	High (#)	3 ± 2	3 ± 2	3 ± 2	2 ± 1 ^a^	3 ± 2	2 ± 1	2 ± 1	3 ± 2
	Moderate (#)	10 ± 4	9 ± 4	9 ± 4	9 ± 4	9 ± 3	8 ± 5	9 ± 3	9 ± 2
**#_DEC_**	High (#)	4 ± 2	3 ± 2 ^a^	3 ± 2 ^a^	2 ± 2 ^a^	3 ± 2	3 ± 1	2 ± 3 ^a^	2 ± 2
	Moderate (#)	5 ± 2	5 ± 3	5 ± 3	5 ± 2	4 ± 2	5 ± 3	4 ± 2	4 ± 2

ACCdist: Acceleration distance, AU: Arbitrary units, DECdist: Deceleration distance, HSR: High-speed running, LSR: Low-speed running, MSR: Moderate-speed running, PL: Player Load, TD: SPR: Sprinting, Total Distance, #_ACC_: Number of accelerations, #_DEC_: Number of decelerations,

^a^ different from 0–5 min at p≤0.05 level,

^b^ different from 0–5 min at p≤0.001 level.

**Table 5:** Magnitude of change in physical performance variables for substitutes from timing of pitch-entry to the end of match-play.

Variable		0–5 min (*n = 33*)	5–10 min (*n = 32*)	10–15 min (*n = 30*)	15–20 min (*n = 26*)	20–25 min (*n = 19*)	25–30 min (*n = 11*)	30–35 min (*n = 7*)	35–40 min (*n = 4*)	Time effects	Position effects	Scoreline effects
**TD**	Absolute (m)	REF	-71.76 (-102.23 to -41.31)^b^	-73.29 (-104.32 to -42.27)^b^	-66.61 (-98.96 to -34.26)^b^	-71.39 (-107.16 to -35.61)^b^	-91.22 (-134.72 to -47.70)^b^	-92.98 (-145.39 to -40.57)^b^	-84.91 (-151.78 to -18.04)^a^	-11.30 (-16.97 to -5.63)**	MID˃ATT** MID˃DEF*	WI˃DR* WI˃LO**
	Relative (m∙min^-1^)	REF	-14.58 (-20.70 to -8.46)^b^	-14.40 (-20.64 to -8.17)^b^	-13.62 (-20.12 to -7.12)^b^	-14.46 (-21.65 to -7.28)^b^	-18.26 (-27.01 to -9.51)^b^	-18.64 (-29.17 to -8.11)^b^	-16.45 (-29.90 to -3.00)^a^	-2.28 (-3.14 to -1.14)**	MID˃ATT** MID˃DEF*	WI˃DR* WI˃LO**
**LSR**	Absolute (m)	REF	-23.31 (-47.66 to 1.04)	-23.20 (-48.02 to 1.62)	-23.14 (-49.05 to 3.10)	-35.58 (-64.25 to -6.93)^a^	-31.80 (-66.70 to 3.10)	-16.07 (-58.11 to 25.97)	-16.07 (-69.20 to 38.04)	n/a	MID˃ATT*	WI˃DR*
	Relative (m∙min^-1^)	REF	-4.59 (-9.36 to 0.16)	-4.63 (-9.48 to 0.21)	-4.75 (-9.81 to 0.30)	-7.05 (-12.65 to -1.46)^a^	-6.14 (-12.95 to 0.67)	-3.06 (-11.26 to 5.14)	-2.44 (-12.92 to 8.04)	n/a	MID˃ATT*	WI˃DR*
**MSR**	Absolute (m)	REF	-32.61 (-47.06 to -18.16)^b^	-27.30 (-41.99 to -12.59)^b^	-26.31 (-41.60 to -11.03)^b^	-21.05 (-37.87 to -4.21)^a^	-38.47 (-58.82 to -18.12)^b^	-49.65 (-74.12 to -25.18)^b^	-34.21 (-65.41 to -3.01)^a^	-4.37 (-7.01 to -1.74)**	MID˃ATT* MID˃DEF*	n/a
	Relative (m∙min^-1^)	REF	-6.52 (-9.41 to -3.63)^b^	-5.45 (-8.40 to -2.52)^b^	-5.26 (-8.32 to -2.21)^b^	-4.21 (-7.58 to -0.84)^a^	-7.69 (-11.76 to -3.62) ^b^	-9.93 (-14.82 to -5.04)^b^	-6.84 (-13.08 to -0.60)^a^	-4.37 (-7.01 to -1.74)**	MID˃ATT* MID˃DEF**	n/a
**HSR**	Absolute (m)	REF	-19.70 (-28.85 to -10.56)^b^	-23.23 (-32.55 to -13.91)^b^	-21.03 (-30.75 to -11.30)^b^	-14.30 (-25.06 to -3.55)^a^	-25.50 (-38.59 to -12.41)^b^	-29.20 (-44.97 to -13.42)^b^	-33.23 (-53.33 to -13.12)^b^	-3.38 (-5.10 to -1.65)**	MID˃DEF*	n/a
	Relative (m∙min^-1^)	REF	-3.94 (-5.77 to -2.11)^b^	-4.65 (-6.51 to -2.78)^b^	-4.21 (-6.15 to -2.26)^b^	-2.86 (-5.01 to -0.71)^a^	-5.10 (-7.72 to -2.48)^b^	-5.84 (-8.99 to -2.68)^b^	-6.65 (-10.66 to -2.62)^b^	-0.68 (-1.02 to -0.33)**	MID˃DEF*	n/a
**SPR**	Absolute (m)	REF	3.83 (-1.38 to 9.04)	0.30 (-5.01 to 5.61)	3.63 (-1.91 to 9.17)	-1.04 (-7.17 to 5.08)	3.96 (-3.51 to 11.43)	-0.19 (-9.18 to 8.80)	-4.15 (-15.64 to 7.33)	n/a	n/a	n/a
	Relative (m∙min^-1^)	REF	0.77 (-0.28 to 1.81)	0.06 (-1.00 to 1.81)	0.73 (-0.38 to 1.83)	-0.21 (-1.43 to 1.02)	0.79 (-0.70 to 2.29)	-0.04 (-1.84 to 1.76)	-0.83 (-3.13 to 1.47)	n/a	n/a	n/a
**PL**	Absolute (AU)	REF	-7.38 (-10.65 to -4.12)^b^	-8.49 (-11.83 to -5.16)^b^	-7.97 (-11.45 to -4.49)^b^	-7.60 (-11.45 to -3.74)^b^	-10.61 (-15.31 to -5.91)^b^	-12.56 (-18.23 to -6.89)^b^	-13.46 (-20.68 to -6.23)^b^	-1.67 (-2.29 to -1.06)**	n/a	WI˃LO*
	Relative (AU∙min^-1^)	REF	-1.50 (-2.16 to -0.85)^b^	-1.68 (-2.35 to 1.01)^b^	-1.62 (-2.32 to -0.92)^b^	-1.55 (-2.33 to -0.78)^b^	-2.13 (-3.08 to -1.19)^b^	-2.53 (-3.66 to -1.39)^b^	-2.70 (-4.15 to -1.25)^b^	-0.34 (-0.46 to -0.21)**	n/a	WI˃LO*
**_ACC_dist**	High (m)	REF	-1.33 (-2.68 to 0.00)^a^	-2.48 (-3.85 to -1.11)^b^	-3.05 (-4.47 to -1.62)^b^	-0.61 (-2.19 to 0.96)	-1.54 (-3.46 to 0.37)	-4.03 (-6.34 to -1.73)^b^	-1.60 (-4.53 to 1.33)	-0.27 (-0.53 to -0.02)*	n/a	WI˃DR* WI˃LO**
	Moderate (m)	REF	-3.22 (-5.03 to -1.41)^b^	-3.62 (-5.47 to -1.77)^b^	-3.59 (-5.52 to -1.67)^b^	-2.54 (-4.67 to -0.42)^a^	-3.80 (-6.37 to -1.22)^a^	-5.57 (-8.67 to -2.47)^b^	-4.74 (-8.69 to -0.80)^a^	-0.55 (-0.88 to -0.22)**	MID˃DEF*	WI˃LO*
**_DEC_dist**	High (m)	REF	-1.24 (-2.29 to -0.18)^a^	-1.84 (-2.91 to -0.76)^a^	-0.93 (-2.05 to 0.19)	-0.30 (-1.54 to 0.94)	-1.75 (-3.26 to -0.24)^a^	-2.17 (-3.98 to -0.35)^a^	-2.40 (-4.72 to -0.08)^a^	n/a	MID˃DEF**	WI˃LO**
	Moderate (m)	REF	-1.39 (-2.89 to 0.11)	-1.56 (-3.09 to -0.03)^a^	-0.93 (-2.53 to 0.66)	-0.53 (-2.30 to 1.24)	-1.40 (-3.57 to 0.76)	-2.47 (-5.35 to 0.14)	-2.02 (-5.35 to 1.31)	n/a	MID˃DEF**	WI˃DR* WI˃LO**
**#_ACC_**	High (IRR)	REF	0.98 (0.74 to 1.29)	0.87 (0.65 to 1.16)	0.66 (0.47 to 0.92)^a^	1.11 (0.81 to 1.52)	0.74 (0.48 to 1.15)	1.01 (0.54 to 1.89)	0.76 (0.56 to 1.02)	n/a	MID˂ATT*	WI˃DR* WI˃LO*
	Moderate (IRR)	REF	0.94 (0.80 to 1.10)	0.88 (0.74 to 1.04)	0.88 (0.74 to 1.05)	0.88 (0.73 to 1.06)	0.83 (0.66 to 1.06)	0.98 (0.74 to 1.30)	0.96 (0.67 to 1.39)	n/a	n/a	n/a
**#_DEC_**	High (IRR)	REF	0.70 (0.53 to 0.92)^a^	0.69 (0.52 to 0.92)^a^	0.66 (0.49 to 0.89)^a^	0.90 (0.67 to 1.22)	0.68 (0.45 to 1.03)	0.54 (0.31 to 0.93)^a^	0.57 (0.29 to 1.13)	n/a	MID˃ATT* MID˃DEF*	WI˃LO*
	Moderate (IRR)	REF	0.95 (0.75 to 1.19)	1.05 (0.84 to 1.31)	1.01 (0.80 to 1.29)	0.91 (0.69 to 1.19)	1.06 (0.78 to 1.45)	0.75 (0.48 to 1.15)	0.78 (0.46 to 1.36)	n/a	n/a	WI˃DR* WI˃LO**

ACCdist: Acceleration distance, ATT = Attacker, AU: Arbitrary units, DECdist: Deceleration distance, DEF = Defender, DR: Scores level at the time of pitch-entry, HSR: High-speed running, LO: Team losing at the time of pitch-entry, LSR: Low-speed running, MID: Midfielder, MSR: Moderate-speed running, PL: Player Load, REF: Reference category for comparison, SPR: Sprinting, TD: Total Distance, WI: Team winning at the time of pitch-entry, #_ACC_: Number of accelerations, #_DEC_: Number of decelerations,

^a^ different from 0–5 min at p≤0.05 level when ‘epoch’ modelled as categorical,

^b^ different from 0–5 min at p≤0.001 level when ‘epoch’ modelled as categorical,

*: Significant effect at p≤0.05,

**: Significant effect at p≤0.001.

Data are reported as effect estimates (95% CI) except for #_ACC_ and #_DEC,_ which are incidence risk ratios (IRR).

When compared with midfielders, attackers and defenders covered less TD (-41.22, CI: -63.68 to -18.77 m, and -41.15, CI: -74.48 to -7.82 m), MSR (-14.57, CI: -26.49 to -2.64 m, and -28.36, CI: -45.21 to -11.51 m), and performed fewer #H_DEC_ (IRR: 0.77, CI: 0.64 to 0.93, and 0.70, CI: 0.52 to 0.95) per five min epoch. Moreover, defenders covered less HSR (-19.95, CI: -33.21 to -6.70 m), M_ACC_dist (-3.20, CI: -5.46 to -0.93 m), H_DEC_dist (-1.88, CI: -3.02 to -0.75 m), and M_DEC_dist (-3.62, CI: -5.48 to -1.76 m), whilst attackers executed more #H_ACC_ (IRR: 1.25, CI: 1.04 to 1.50) than midfielders. When the team was drawing or losing at the time of pitch-entry, players covered less TD (-26.64, CI: -52.45 to -0.83 m, and -48.71, CI: -75.40 to -22.02 m), H_ACC_dist (-1.89, CI: -3.08 to -0.69 m, and -2.44, CI: -3.74 to -1.14 m), and M_DEC_dist (-1.48, CI: -2.75 to -0.20 m, and -3.48, CI: -4.85 to -2.10 m), in addition to performing fewer #H_ACC_ (IRR: 0.79, CI: 0.64 to 0.97, and 0.80, CI: 0.65 to 0.99) and #M_DEC_ (IRR: 0.82, CI: 0.67 to 0.99, and 0.69, CI: 0.56 to 0.84) per five min epoch, compared with when the team was winning. Moreover, substitutes introduced when the scores were level accumulated less LSR (-26.00, CI: -49.75 to -2.25 m), whilst players entering the pitch when the reference team was losing performed less M_ACC_dist (-2.03, CI: -3.70 to -0.36 m), H_DEC_dist (-1.83, CI: -2.74 to -0.93 m), and fewer #H_DEC_ (IRR: 0.76, CI: 0.61 to 0.94) per five min epoch, alongside returning lower PL values (-5.10, CI: -8.53 to -1.68 AU), when compared with when the team was winning at the time of introduction.

## Discussion

The aim of this study was to investigate the match-day physical demands experienced by English Championship soccer substitutes before and after pitch-entry. Prior to introduction into a match, substitutes performed 3±1 rewarm-up bouts∙player^-1^∙match^-1^, with rewarm-ups becoming shorter and more intense (i.e., increasing relative TD, LSR, MSR, and HSR ∙bout^-1^) as pitch-entry approached. Following introduction, time negatively influenced absolute and relative TD, MSR, HSR, and PL, as well as H_ACC_dist, and M_ACC_dist. Playing position and match scoreline also influenced the movement profiles observed, with the greatest match-distances being covered by midfielders (i.e., compared with attackers and defenders), and on occasions when the team was winning at the time of introduction (i.e., compared with when drawing or losing). Such data provide novel insights into transient changes in the match-day movement demands experienced by substitutes from a professional soccer club and highlight important future research opportunities, findings of which have the potential to positively influence practitioners seeking to optimise the match-day strategies for this bespoke population of soccer players.

Substitutes covered ~37.9 m∙min^-1^ during their initial warm-up, of which 97% was LSR (Table 2), and then performed ~3 subsequent rewarm-ups prior to pitch-entry. Acknowledging that other non-pitch-based actions may also have occurred, this study provides potentially important observations regarding the frequency and/or intensity (HSR: 0–2 m∙min^-1^ during each bout) of pre-entry activities in professional soccer players. Whilst the absence of a comparator trial limits our ability to comment on the suitability of this pattern of activity for subsequent match-play, increasing the intensity of warm-up exercise from 300 m of striding (6 x 50 m) to an equidistant bout of combined striding (100 m) and race-pace running (200 m) has been shown to enhance subsequent 800 m running performance by ~1% [20]. Moreover, a positive relationship exists between body temperature increases and performance in tasks requiring high-velocity muscle actions, with improvements of ~2–10% being reported for every 1°C increase [21]. Rapid declines in core (T_core_) and muscle (T_m_) temperature alongside concomitant reductions in physical performance occur following the cessation of exercise, with body temperature returning to baseline within 15–20 min in ambient conditions (10–30°C: [22, 23]). Indeed, better maintenance of body temperature and improved physical performance capacity has been demonstrated when rewarm-up activity (e.g., low and medium-intensity aerobic exercise, intermittent agility exercise, resistance exercise, whole-body vibration etc.) is performed during a ~15 min half-time interval, when compared with the responses observed following the equivalent period of passive rest [24–29]. Moreover, consistent with observations that increasing warm-up intensity may be beneficial for subsequent high-intensity exercise performance [20], where ≥15 min separates an initial warm-up and entry into a match, active rewarm-ups consisting of brief high-intensity efforts (~2 min at ~90% HR_max_) may better maintain subsequent performance in explosive tasks relative to passive rest [22]. Substitutes in the current study covered ~29.1 m∙min^-1^ (including ~0.4 m∙min^-1^ of HSR) during the 15 min prior to pitch entry but notably no sprinting occurred in either the initial warm-up or subsequent rewarm-ups (Table 2). Whilst the efficacy of this pre pitch-entry strategy as a means of preparing for subsequent performance remains to be determined, combining modified rewarm-up strategies with passive heat maintenance techniques (e.g., wearing specialist heat-retaining garments) could further assist in preservation of body temperature and optimised performance thereafter (i.e., before pitch-entry: [22, 23, 26, 27]).

Given the time-frames involved, and the desire to maintain energy stores, it may be suggested that optimising rewarm-up strategies is of greater importance to substitutes than is the initial warm-up. In English soccer, coaches are not permitted to leave the ‘technical area’ whilst the match is underway, therefore the content and intensity of rewarm-ups is likely determined primarily by the players themselves in the absence of a practiced routine. As superior outcomes are reported as a result of coach-supervised versus unsupervised training [30], such regulations may negatively impact upon the quality of preparatory activity undertaken. Indeed, empirical evidence highlighted that events unfolding on the pitch (such as the proximity of match-play to the rewarm-up area) directly influenced the activities being performed by substitutes. Interestingly, although the scoreline at the point of pitch-entry does not necessarily reflect the scoreline at the time of any given pre-entry rewarm-up, players performed more HSR per rewarm-up bout (+3.17 m) when the team was losing at the time of introduction, than when compared with being ahead. Whilst the adequacy of the pre pitch-entry regimes of the players sampled remains to be determined, it is plausible that more structured rewarm-up protocols, the presence of additional personnel (e.g., coaching staff), and/or the provision of larger rewarm-up spaces (that may facilitate sprinting) and/or equipment may influence the preparatory actions undertaken before pitch-entry, thus affecting on-pitch performance thereafter. Notably, in addition to allowing a fourth substitution to be made in matches progressing to extra-time, regulations at the 2018 Fédération Internationale de Football Association (FIFA) World Cup finals permitted up to six substitutes at a time to be accompanied by two coaches in a designated rewarm-up area behind the goalposts [31].

Observations of decreases in relative running distances following the substitutes’ initial 5 min of match-play (Tables 4 and 5) contradict previous reports of a trend towards increasing TD and HSR for English Premier League substitutes as a match progressed [3]. Indeed, our findings better aligned with suggestions that whilst starting players may adopt a ‘slow-positive’ pacing strategy in which they conserve energy in an effort to minimise the magnitude of performance decrements over the course of 90 min, the shorter playing duration and desire to make an impact on the match, means that substitutes may favour an ‘all-out’ approach; initially performing at an unsustainable high intensity, followed by an inevitable reduction [32]. Whilst discrepancies may appear to exist, the study in the English Premier League [3] analysed data according to five min match-epochs which were fixed relative to the time of kick-off as opposed to the timing of pitch-entry. This approach may have underestimated the initial demands via omission of data collected in the stages of match-play immediately post-entry.

Relative TD (-12.2%) and HSR (-38.6%) declined substantially between 0–5 min and 5–10 min, but values for the next four epochs (i.e., 5–25 min post-entry) remained within 1 m∙min^-1^ of each other. Speculatively, this performance profile indicates that mechanisms other than either progressive or transient fatigue may explain the findings. The initial ~15 min of soccer match-play elicits the highest intensity in terms of movement demands [3, 6], and these data suggest that such heightened responses may be specific to the timing of match-introduction for any given individual as opposed to the proximity to kick-off per se. Alternatively, players’ own concerns surrounding the lack of opportunity and motivation to prepare for match-play when selected as substitutes [4, 33], highlight that the higher exercise intensity adopted during the 0–5 min following introduction may partially represent an effort, consciously or subconsciously, to account for perceived inadequacies in pre-entry preparation by ‘warming-up’ (i.e., eliciting the physiological responses desired from a pre-performance warm-up) having already entered the field of play. Unfortunately, it cannot be determined whether the movement responses observed immediately following introduction reflected positive match-contributions, or simply a heightened period of activity that did not enhance team performance. Moreover, as discrete five min epochs were employed in this study, albeit normalised on an individual basis to pitch-entry rather than kick-off, it was not possible to determine the exact time-course of this transient increase in running intensity.

As is the case for whole-match players, replacement midfielders covered greater relative distances than players in other positions [6, 12, 34], and the ~101–120 m∙min^-1^ covered following introduction broadly parallels values (~120 m∙min^-1^) reported for substitutes in the English Premier League [3]. Moreover, relative HSR distance (~4–10 m∙min^-1^) also reflected these previous data [3]. However, the mean running demands reported by Bradley et al. [3] correspond approximately to the values observed for 0–5 min in the current study, with values for subsequent epochs showing marked declines in comparison (TD: 101–106, HSR: 4–7 m∙min^-1^). Such discrepancies may be attributable to differences in match demands between playing-levels [6], inconsistent methodologies between studies, varying degrees of pre-match preparation, or the influence of situational factors such as the playing ‘style’ of the reference team and/or opposition [14]. Indeed, the potential influence of contextual factors is highlighted in the current study by the differing movement demands according to the match scoreline at the time of pitch-entry. Such responses may reflect changes in the ‘momentum’ of a match, tactical or strategic objectives (e.g., playing ‘style’), and/or the relative quality of the reference team and their opponents [14].

Acknowledging that the present data are derived from one team during one season, and that MEMS may be incapable of detecting every aspect of substitutes’ individualised pre-entry preparation (e.g., less dynamic activities such as stretching, cycling etc.), the movement profiles observed in this study highlight a number of avenues for further exploration; the findings of which may aid practitioners seeking to optimise match-day preparatory strategies. Indeed, research into the efficacy of pre pitch-entry interventions for substitute players, such as modifying active rewarm-up practices and/or employing passive heat maintenance techniques whilst awaiting introduction, alongside consideration of nutritional strategies (e.g., consuming caffeinated chewing gum), is warranted. The pre-performance period has also been identified as an opportunity to enhance hormonal and psychological responses [21, 35]. Self-motivation may enhance subsequent performance [21], and watching video footage of players’ own previous success has been associated with elevated free testosterone concentrations and improved measures of overall match-performance thereafter [35]. Such observations highlight a potential role for strategies which may positively contribute to manipulating the environment in which substitutes await pitch-entry.

The execution of technical and/or tactical skills is an important component of soccer performance [10, 36], and tactical motivations may underpin a large proportion of substitutions made [4]. In addition to the lack of technical/tactical information, MEMS indices alone cannot quantify substitutes’ overall contribution to a match. Alongside key physiological measurements such as body temperature responses, research combining MEMS data with analysis of the match-consequences of any periods of heightened activity may allow further commentary on the potential reasons underlying the movement patterns observed following pitch-entry. Moreover, future research should analyse substitutes’ match performance with reference to their tactical impact (e.g., changing team formation or ‘style’), whilst noting the precise reasons for their introduction. All of the substitutes in the current study were introduced to play in their preferred tactical position. However, given the differences in match demands experienced by different playing positions, it is plausible that the responses may differ in instances where a player is required to adopt an unfamiliar tactical role. Finally, given that soccer teams may adopt a ‘rotation policy’ in which the use of substitutes represents an attempt to minimise the accumulation of fatigue across a competition period [4], investigation into the magnitude of the post-match fatigue response for substitute players, who face unique match-day demands, may aid practitioners in tailoring training and/or recovery strategies.

## Conclusions

Acknowledging that these observations are based upon one team, and that other soccer clubs may adopt different pre-entry practices, substitutes in the current study performed ~3 bouts of rewarm-up activity prior to entering the pitch, with increases in relative TD and PL, but decreasing rewarm-up duration as pitch-entry approached. Considering existing recommendations for the structure of warm-ups in team sports [22], alongside the time-course of body temperature changes [21, 23, 27], further research is required to examine whether the observed strategies are adequate to prepare players for optimal performance upon pitch-entry. Such investigations may be conducted through the use of soccer simulation protocols, which allow assessment of physiological and performance responses without the confounding influence of many of the situational variables inherent in soccer match-play [37]. Although substitutes performed at substantially higher intensities during the 0–5 min following introduction compared with 5–10 min, the underlying reasons and match-consequences of the observed responses remain unclear.
